# Autophagy maturation associated with CD38-mediated regulation of lysosome function in mouse glomerular podocytes

**DOI:** 10.1111/jcmm.12173

**Published:** 2013-11-17

**Authors:** Jing Xiong, Min Xia, Ming Xu, Yang Zhang, Justine M Abais, Guangbi Li, Christopher R Riebling, Joseph K Ritter, Krishna M Boini, Pin-Lan Li

**Affiliations:** Department of Pharmacology and Toxicology, Virginia Commonwealth University School of MedicineRichmond, VA, USA

**Keywords:** CD38, autophagic flux, glomeruli, ADP-ribosylcyclase, lysosome trafficking

## Abstract

Podocytes are highly differentiated glomerular epithelial cells that contribute to the glomerular barrier function of kidney. A role for autophagy has been proposed in maintenance of their cellular integrity, but the mechanisms controlling autophagy in podocytes are not clear. The present study tested whether CD38-mediated regulation of lysosome function contributes to autophagic flux or autophagy maturation in podocytes. Podocytes were found to exhibit a high constitutive level of LC3-II, a robust marker of autophagosomes (APs), suggesting a high basal level of autophagic activity. Treatment with the mTOR inhibitor, rapamycin, increased LC3-II and the content of both APs detected by Cyto-ID Green staining and autophagolysosomes (APLs) measured by acridine orange staining and colocalization of LC3 and Lamp1. Lysosome function inhibitor bafilomycin A1 increased APs, but decreased APLs content under both basal and rapamycin-induced conditions. Inhibition of CD38 activity by nicotinamide or silencing of CD38 gene produced the similar effects to that bafilomycin A1 did in podocytes. To explore the possibility that CD38 may control podocyte autophagy through its regulation of lysosome function, the fusion of APs with lysosomes in living podocytes was observed by co-transfection of GFP-LC3B and RFP-Lamp1 expression vectors. A colocalization of GFP-LC3B and RFP-Lamp1 upon stimulation of rapamycin became obvious in transfected podocytes, which could be substantially blocked by nicotinamide, CD38 shRNA, and bafilomycin. Moreover, blockade of the CD38-mediated regulation by PPADS completely abolished rapamycin-induced fusion of APs with lysosomes. These results indicate that CD38 importantly control lysosomal function and influence autophagy at the maturation step in podocytes.

## Introduction

Podocytes are highly specialized epithelial cells that compose the renal filtration barrier, but are considered among the most vulnerable cell types of the kidney. Their loss or dysfunction is thought to be critical for the pathogenesis of proteinuria and glomerular disease. As podocytes are terminally differentiated, non-dividing cells with a low capacity for regeneration (37), the mechanisms that contribute to their adaptive responses to stress or injury are likely to play a critical role in their physiology. Recent evidence supports an important role of autophagy in the differentiation of podocytes and in glomerular pathologies (5). Autophagy-deficient mice exhibited significantly greater elevations in serum urea nitrogen and creatinine, compared with control animals 1. Podocyte-specific deletion of a gene coding autophagy protein 5 (*Atg5*) led to a glomerulopathy in ageing mice that was accompanied by an accumulation of oxidized and ubiquitinated proteins, endoplasmic reticulum (ER) stress, and proteinuria, which ultimately resulted in podocyte loss and late-onset glomerulosclerosis 2. Thus, it is important to explore the mechanisms involved in autophagy in podocytes in order to prevent podocyte damage induced by the defective autophagy.

Autophagy is a cell survival mechanism that contributes to the degradation of long-lived or damaged proteins and excessive or dysfunctional cell organelles 3,4. Under normal physiological conditions, autophagy functions in a continuous, reparative way to maintain normal cellular homeostasis, whereas under conditions of stress or pathological stimuli, it may be induced and even develop into autophagic death. Thus, autophagy is a cellular process of ‘self-eating’ whereby cytoplasmic components are sequestered into autophagic vesicles or vacuoles [called autophagosomes (APs)] and then delivered to lysosomes for degradation 6,7. Recent research has delineated an evolutionarily conserved molecular machinery of autophagy, which includes a specific class of genes or proteins called autophagy-related genes, Atgs 6,7. Atgs interact with each other and other regulatory proteins to form various protein complexes for the initiation, expansion, and final maturation of APs. Deficiency of a specific Atg leads to inhibition of relevant autophagic events. The process of autophagy occurs in four distinct stages including induction, APs formation, docking and fusion with lysosomes to form autophagolysosomes (APLs), and breakdown of autophagic vesicles 9–10. The formation of APs and related regulation are relatively well-understood 11, but the molecular mechanisms that contribute to their fusion with lysosomes are still poorly understood.

In this regard, intracellular Ca^2+^ has been reported to be involved in the regulation of autophagy 12–15, and lysosome fusion to the cell plasma membrane, endosome, phagosome, AP and other organelles was shown to be Ca^2+^-dependent 5–17. Furthermore, inhibition of lysosome function by bafilomycin A1, an inhibitor of the vacuolar H^+^ pump, resulted in the failure of Ca^2+^ to mobilize from lysosomes and a blockade of autophagy 18. More recently, our laboratory has reported that lysosome function was fine controlled by a CD38-ADP-ribosylcylase-mediated signalling pathway, which is mainly attributed to its enzymatic product, nicotinic acid adenine dinucleotide phosphate (NAADP). Nicotinic acid adenine dinucleotide phosphate is a most potent intracellular Ca^2+^ mobilizer and lysosome function regulator *via* its action on lysosome Ca^2+^ bursts 19–20. It remains unknown whether this CD38-mediated regulation of lysosome function is involved in the autophagic process, in particular, in autophagy maturation. It has been suggested in the present study that lysosome function including its trafficking and fusion is well-controlled by CD38-NAADP signalling pathway *via* its action to induce lysosomal Ca^2+^ bursts in podocytes and such regulation of lysosome function is critical for autophagy maturation. To test this hypothesis, we first characterized autophagic process including the formation of APs and autophagic flux in podocytes. We then examined whether CD38-mediated regulation of lysosome function such as it’s trafficking or fusion is involved in autophagy maturation in podocytes.

## Materials and methods

### Cell culture

Conditionally immortalized mouse podocytes (kindly provided by Dr. Paul Klotman, Division of Nephrology, Department of Medicine, Mount Sinai School of Medicine, New York, NY, USA) were cultured at 33°C on collagen I-coated flasks or plates in RPMI 1640 medium supplemented with recombinant mouse interferon–γ. The cells were then differentiated by culturing at 37°C for 10–14 days in medium without interferon–γ prior to their use in experiments. For nutrient starvation 21, podocytes were incubated in Earle’s balanced salt solution (EBBS; Sigma-Aldrich, St. Louis, MO, USA) for 90 min.

### RNA interference of CD38 gene expression

A plasmid encoding a CD38 shRNA directed at the target sequence 5′-GACAGACCTGGCTGCCGCCTCTCTAGGAA-3′ was purchased from Origene (Rockville, MD, USA). A corresponding plasmid encoding scrambled shRNA was used as a control. The shRNA plasmids were transfected using TransFectin Lipid Reagent (Life Science Research, Bio-Rad, Hercules, CA, USA) according to the manufacturer’s instructions. The effectiveness of the CD38 shRNA in silencing the CD38 gene expression in podocytes 22 was confirmed using RT-PCR, immunoblotting, and immunocytochemistry. We found that CD38 shRNA transfection inhibited the CD38 expression by 50% compared to scramble shRNA transfected cells (Fig. S1).

### Autophagosome assay by flow cytometry

The autophagosomal content of podocytes was assayed using the Cyto-ID Green autophagy detection reagent according to the manufacturer’s instructions (Enzo Life Sciences Inc., Farmingdale, NY, USA). Briefly, podocytes were seeded on to six well plates at 1 × 10^5^ cells/well. Following compound treatment, the cells were washed once in phosphate-buffered saline and stained in medium containing Cyto-ID Green autophagy detection reagent (1 μl/4 ml). After incubation for 30 min. at 37°C, the cells were trypsinized and the cell suspension was analysed by flow cytometry using the green fluorescence channel (FL1) (533/30 nm band-pass filter with excitation at 488 nm) of the flow cytometer (BD Biosciences, San Jose, CA, USA).

### Quantification of autophagic vesicular organelles

Podocytes were seeded on to six well plates at 1 × 10^5^ cells/well. Following compound treatment, the cell monolayers were stained for 20 min. with acridine orange (AO; 5 μl/ml; Sigma-Aldrich), removed from the plate by trypsinization and collected for analysis using a FACS Calibur (Becton Dickinson, Franklin Lakes, NJ, USA). Green (510–530 nm) and red (650 nm) fluorescence emission from 5000 cells illuminated with blue (488 nm) excitation light was measured, and the autophagic vesicular organelles were quantified by the red/green fluorescence ratio for individual cells by using CytoSoft 4.2 software (Guava Technologies, Hayward, CA, USA).

### Immunoblot analysis

Cell lysates were prepared by scraping cells into an ice-cold buffer containing protease inhibitors and measuring the protein (Bio-Rad). Total protein (20 μg) was electrophoresed through a 12% SDS-PAGE gel and transferred onto a nitrocellulose membrane (Immobilon-P; Millipore, Billerica, MA, USA). The membrane was probed with primary antibodies (1:1000 dilution) against LC3 or p62 (Cell Signalling Technology Inc., Danvers, MA, USA) by overnight incubation at 37°C followed by incubation with a horseradish peroxidase-conjugated anti-IgG secondary antibody (1:5000 dilution; Santa Cruz Biotechnology Inc., Santa Cruz, CA, USA). The immunoreactive bands were detected by chemiluminescence (ECL; Amersham Biosciences, Pittsburgh, PA, USA) and visualized on Kodak X-Omat X-ray film. Densitometric analysis of the film images was performed with ImageJ software (NIH, Bethesda, MD, USA).

### Double immunofluorescent staining

Cells were grown on coverslips and fixed in PBS containing 4% paraformaldehyde for 10 min. Donkey serum (5%; Sigma-Aldrich) was included in all blocking and primary and secondary antibody buffers. Coverslips were incubated with primary antibodies to LC3, Lamp-1, ubiquitin or p62 overnight at 4°C. Secondary antibodies were Alexa Fluor-conjugated (Invitrogen Inc., Grand Island, NY, USA). Coverslips were mounted in Vectashield reagent containing DAPI (Vector Laboratories Inc., Burlingame, CA, USA). The colocalization of LC3 and Lamp-1 and ubiquitin and p62 was determined by using a confocal laser scanning microscope (Fluoview 1000; Olympus, Tokyo, Japan). Images were analysed by using Image J software. Pearson’s and Mander’s coefficients were used for assessing colocalization.

### Imaging of autophagy in live cells

Low passage number podocytes were plated onto 35-mm dishes at 4 × 10^4^ cells/dish and transduced at ∼70% confluency with baculoviruses containing GFP-LC3 and RFP-Lamp1 transgenes (BacMan 2.0; Invitrogen Inc.) (12 μl/ml medium). After incubation overnight (≥16 hrs) to allow expression of GFP-LC3B and RFP-Lamp1, the cells were treated and then imaged for GFP and RFP by using confocal fluorescence microscopy.

### Fluorescent microscopic measurement of Ca^2+^ release from lysosomes

Intracellular Ca^2+^ responses to rapamycin or calcium inhibitors were determined by using the Ca^2+^-sensitive fluorescent dye, Fura-2, with a fluorescence imaging system as described previously 23–24. Briefly, podocytes were treated with either vehicle or rapamycin for 24 hrs and then loaded with 10 μM Fura-2 at room temperature for 30 min. following by washing three times with Ca^2+^-free Hanks’ buffer supplemented with 2 μM EGTA. The ratio of Fura-2 emissions after excitation at 340 and 380 nm was monitored by using a ratiometric spectrofluorometric microscope (PTI Inc., Birmingham, NJ, USA) before and after treatment with 200 μM Gly-Phe-β-naphthylamide (GPN), a lysosomotropic agent that causes lysosome-dependent calcium release. To determine the effects of calcium inhibitors on the rapamycin or GPN-induced Ca^2+^ response, podocytes were pre-treated for 30 min. with 50 μM PPADS (pyridoxalphosphate-6-azophenyl-2′,4′-disulfonic acid, an antagonist of NAADP), 50 μM ryanodine (a ryanodine receptor antagonist), or 6.25 μM 2-aminoethoxydiphenyl borate (2-APB, an IP_3_R inhibitor). Metafluor^®^ (Universal Imaging Corporation, Downingtown, PA, USA) imaging and analysis software was used to acquire, digitize and store the images for offline processing and statistical analysis.

### Statistics

All data are presented as the mean ± SEM. Significant differences between and within multiple groups were examined using anova for repeated measures, followed by Duncan’s multiple-range test. A *P* < 0.05 was considered as statistically significant.

## Results

### Autophagosomes formation in podocytes

**Figure 1:**
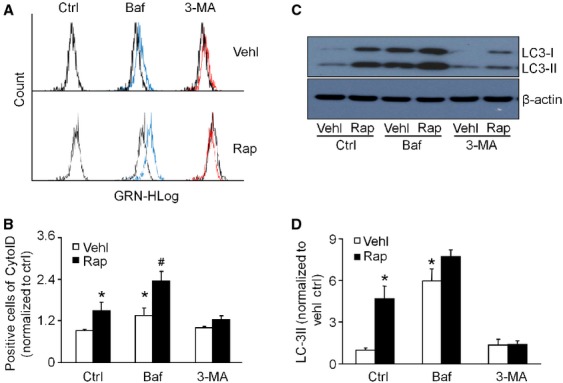
Induction and inhibition of autophagosome formation in podocytes. Podocytes were cultured for 24 hrs with 100 nM rapamycin in the absence or presence of 10 nM bafilomycin A1 or 5 mM 3-MA. (A and B) Flow cytometric analysis of autophagosomes detected by Cyto-ID Green staining. (C) Immunoblot analysis of LC3, a specific marker of autophagosomes. (D) Densitometric quantification of LC3-II signals on immunoblots. Ctrl: Control; Vehl: Vehicle; Rap: rapamycin; Baf: Bafilomycin. The data are expressed as the mean ± SEM and are representative of six independent experiments. * Significant difference (*P* < 0.05) compared to the values from control group, ^#^ Significant difference (*P* < 0.05) compared to the values from rapamycin only group.

Using cultured murine podocytes, we first characterized the autophagy in podocytes. As shown in Figure 1, flow cytometry and western blot analysis demonstrated that rapamycin, an inhibitor of mTOR 25–26 significantly increased the CytoID staining (Fig. 1A and B) and LC3-II protein levels in podocytes compared to control cells (Fig. 1C and D), suggesting that rapamycin treatment caused an accumulation of APs in podocytes. As this effect could be because of either increased formation or suppressed degradation, hence, the effect of bafilomycin, an inhibitor of the vacuolar type H^+^-ATPase which is required for the acidification of lysosomes and fusion of the lysosome with AP 27, was investigated. Treatment with bafilomycin alone increased the CytoID-positive cell number and enhanced the response to rapamycin, suggesting that rapamycin induces the formation of APs in podocytes. In contrast, 3-MA, an inhibitor of the class III phosophoinositide-3 kinases that participate in conversion of LC3-I to LC3-II 28, inhibited the rapamycin-induced increases in CytoID staining and LC3-II protein expression (Fig. 1).

### Induction and inhibition of autophagolysosome maturation in podocytes

**Figure 2:**
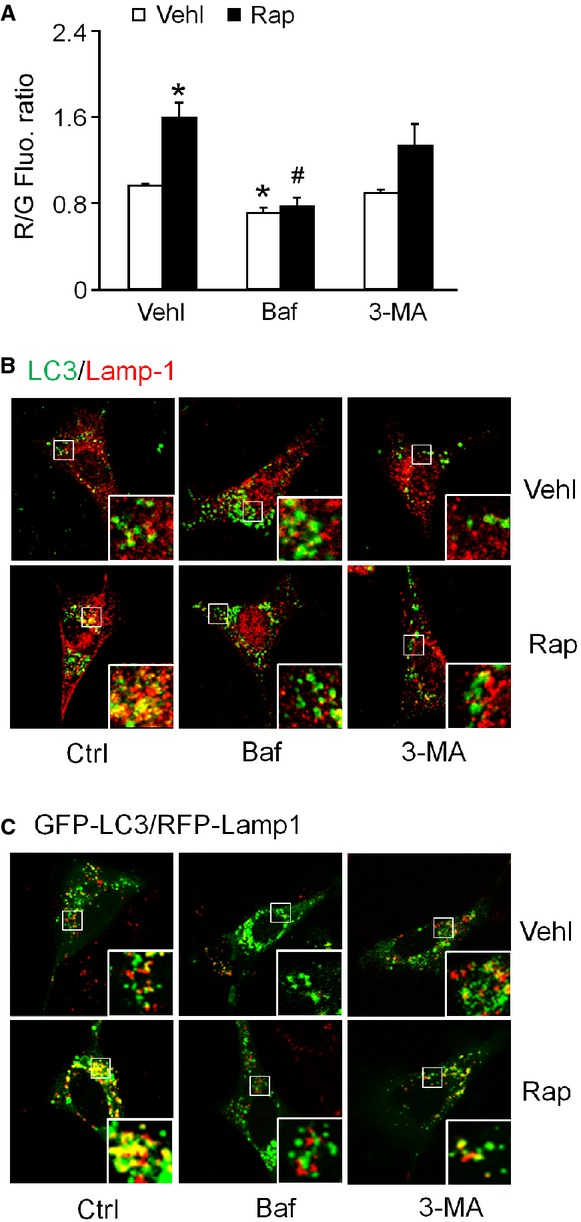
Induction and inhibition of autophagolysosome (APL) maturation in podocytes. Podocytes were cultured for 24 hrs with 100 nM rapamycin in the absence or presence of 10 nM bafilomycin or 5 mM 3-MA. (A) Flow cytometric analysis of the ratio of red/green fluorescence after acridine orange staining, an indicator of the number of APLs. (B) Representative confocal microscopic images of Lamp1 (Alex555, red colour) *versus* LC3 (Alex488, green colour)-stained cells. (C) Podocytes were transiently transfected with GFP-LC3 or RFP-Lamp1 for 24 hrs. Representative images showing the colocalization of GFP-LC3 and RFP-Lamp1 are shown. Ctrl: Control; Vehl: Vehicle; Rap: rapamycin; Baf: Bafilomycin. Data are expressed as the mean ± SEM and are representative of six independent experiments. * Significant difference (*P* < 0.05) compared to the values from control group, ^#^ Significant difference (*P* < 0.05) compared to the values from rapamycin only group.

The maturation of APLs in podocytes was evaluated by flow cytometric analysis of acridine orange staining. As depicted in Figure 2A, rapamycin treatment demonstrated the increased staining with acridine orange, a marker for acidic vesicular organelles. The bafilomycin treatment significantly decreased the acridine orange staining in both vehicle and rapamycin-treated cells, whereas treatment with 3-MA slightly decreased the response to rapamycin (Fig. 2A).

Further double immunofluorescent staining analysis was performed with LC3 and Lamp 1 as markers of APs and lysosomes, respectively, provides a second way to assess the autophagolysosomal content. This approach was applied to both paraformaldehyde-fixed cells stained for detection of endogenous LC3 and Lamp1 protein (Fig. 2B) and in living cells following transduction with GFP-LC3 and RFP-Lamp1 expressing vectors (Fig. 2C). In each case, rapamycin increased the colocalization of LC3 and Lamp1, suggesting an increase in APL content, and this effect was inhibited by bafilomycin and, to a lesser extent, by 3-MA. Bafilomycin increased the green punctuate LC3-associated fluorescence, especially in the GFP-LC3 expressing cells, which is consistent with the accumulation of APs and inhibition of their fusion with lysosomes.

### Inhibitors of autophagy markedly decrease the degradation of p62 and associated ubiquitinated proteins

**Figure 3:**
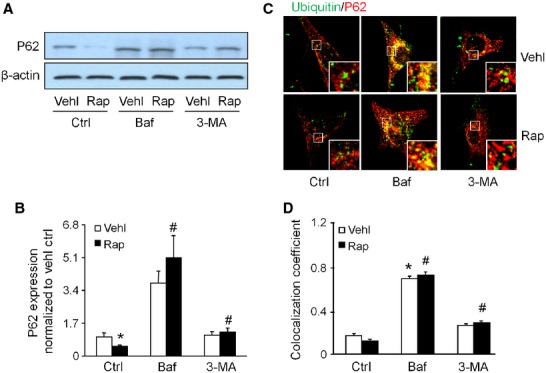
Inhibition of the rapamycin-induced degradation of p62 by autophagy inhibitors in podocytes. Cells were cultured for 24 hrs with vehicle or 100 nM rapamycin in the absence or presence of 10 nM bafilomycin or 5 mM 3-MA. (A) Immunoblot analysis of p62 performed with beta actin as a control. (B) Summarized data from densitometric analysis of the p62 and β-actin signals from immunoblots. (C) Representative confocal microscopic overlaid image of p62 (Alex555, red colour) *versus* ubiquitin (Alex488, green colour) staining. (D) Summarized data showing the colocalization coefficient of p62 and ubiquitin. Ctrl: Control; Vehl: Vehicle; Rap: rapamycin; Baf: Bafilomycin. Data are representative of six independent experiments. Data are expressed as the mean ± SEM. *Significant difference (*P* < 0.05) compared to the values from control group, ^#^ Significant difference (*P* < 0.05) compared to the values from rapamycin only group.

p62 is a polyubiquitin-binding protein associated with autophagosomal membranes which undergoes degradation during autophagy. Thus, the protein levels of p62 are inversely related to autophagic activity 29–30. As shown in Figure 3A and B, rapamycin treatment significantly decreased the P62 protein expression compared to control cells. However, prior treatment with 3-MA and bafilomycin significantly attenuated the rapamycin-induced P62 expression decrease in podocytes. In addition, confocal microscopic analysis demonstrated that colocalization of p62 and ubiquitin was decreased in rapamycin-treated cells, but the bafilomycin and 3-MA treatment attenuated the degradation of APs in vehicle and rapamycin-treated cells (Fig. 3C and D).

### Inhibition of CD38 increased the autophagosomes in podocytes

**Figure 4:**
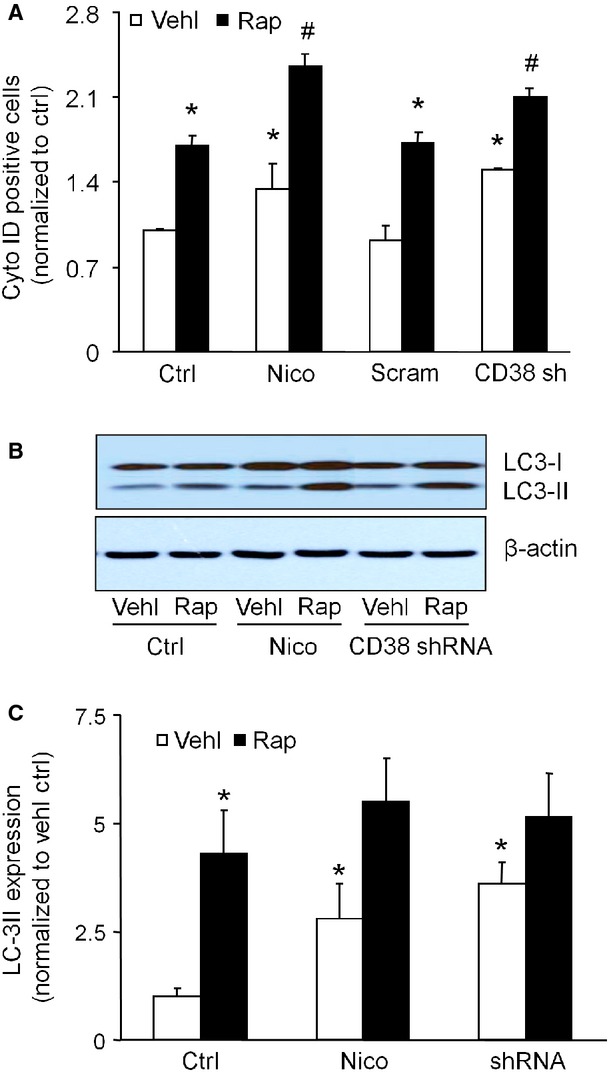
Effects of ADP-ribosylcyclase inhibition and CD38 gene silencing on autophagosome formation in podocytes. Cells were treated for 24 hrs with or without 100 nM rapamycin in the absence or presence of 6 mM nicotinamide (Nico) or transfected CD38 shRNA. (A) Flow cytometric analysis of autophagosomes by CytoID Green staining. (B) Immunoblot analysis of LC3. (C) Densitometric analysis of the LC3-II signal on immunoblots. Ctrl: Control; Vehl: Vehicle; Rap: rapamycin; Nico: Nicotinamide. The data are expressed as the mean ± SEM and are representative of six separate experiments. * Significant difference (*P* < 0.05) compared to the values from control group, ^#^ Significant difference (*P* < 0.05) compared to the values from rapamycin only group.

To further determine the role of CD38/ADP-ribosylcyclase signalling pathway in the regulation of autophagy in podocytes, both pharmacologic and genetic approaches were used. Treatment with the ADP-ribosylcyclase inhibitor, nicotinamide or CD38 shRNA transfection significantly increased the numbers of CytoID- positive cells in control and rapamycin-treated cells (Fig. 4A). As shown in Figure 4B and C, CD38shRNA or nicotinamide treatment significantly increased the LC3-II expression compared to control cells. Rapamycin treatment significantly increased the LC3-II expression compared to control cells, but it had no further effect on nicotinamide or CD38 shRNA transfected cells. Taken together, these data suggest that inhibition of CD38 results in accumulation of APs in podocytes under both the basal condition and following induction of autophagy by rapamycin.

### Lack of lysosome fusion to autophagosomes upon CD38 inhibition

**Figure 5:**
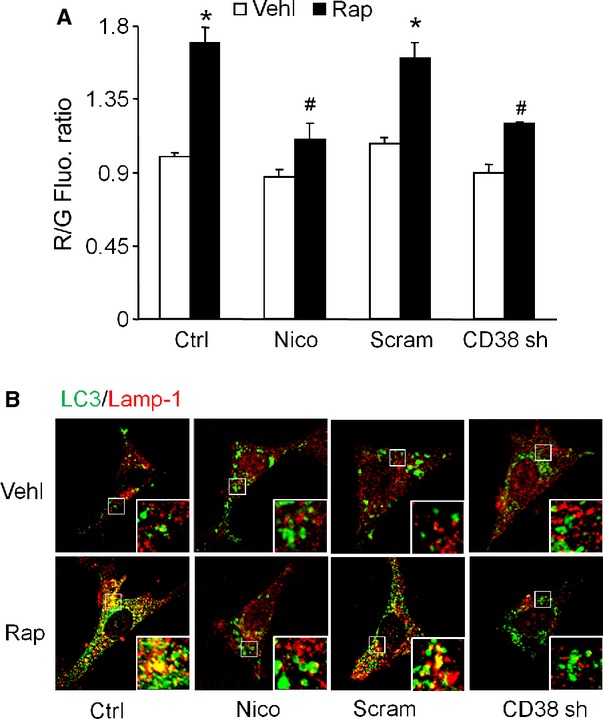
Inhibition of the fusion step of autophagy after ADP-ribosylcyclase inhibition or CD38 gene silencing in podocytes. Podocytes were cultured for 24 hrs with 100 nM rapamycin in the absence or presence of 6 mM nicotinamide or CD38 shRNA transfection. (A) Flow cytometric analysis of the red/green fluorescence ratio after acridine orange staining. (B) Representative confocal microscopic images of Lamp1 (Alex555, red colour) *versus* LC3 (Alex488, green colour) staining. (C) Podocytes were transiently transfected with GFP-LC3 or RFP-Lamp1 for 24 hrs. Representative images of colocalization of GFP-LC3 and RFP-Lamp1 were shown. Ctrl: Control; Vehl: Vehicle; Rap: rapamycin; Nico: Nicotinamide. Data are expressed as the mean ± SEM and are representative of six separate experiments. * Significant difference (*P* < 0.05) compared to the values from control group, ^#^ Significant difference (*P* < 0.05) compared to the values from rapamycin only group.

By using flow cytometry and confocal microscopy, we determined the effect of nicotinamide or CD38 shRNA on markers of autophagolysosomal content in control and rapamycin-treated podocytes. As show in Figure 5, the rapamycin treatment signinificantly increased the acridine orange staining compared to control cells. However, the prior treatment with nicotinamide or inhibition of CD38 significantly attenuated the rapamycin-induced increase in acridine staining in podocytes (Fig. 5A), suggesting that CD38 inhibition reduced the accumulation of APLs upon rapamycin treatment. Furthermore, the evidence of lysosome fusion to AP was also observed by confocal microscopy. Confocal microscopic analysis demonstrated the evidence of a decrease in rapamycin-induced colocalization of LC3B and Lamp1 (shown in yellow patches in Fig. 5B) in podocytes treated with either nicotinamide or CD38 shRNA. The increase in LC3B green fluorescence (Fig. 5B) also supports the conclusion that these treatments increase the autophagosomal content. Similar conclusions were apparent when living cells expressing virus-transduced GFP-LC3 and RFP-Lamp1 were analysed (data was not shown). In addition using starvation as another model of autophagy induction 21, we found that nutrient starvation significantly increased the acridine orange staining (R/G ratio) compared to control cells. However, the prior treatment with nicotinamide significantly attenuated the starvation-induced increase in acridine orange staining in podocytes (Fig. S2). These data suggest that diminished CD38 expression and function blunt the fusion of lysosomes to APs.

### Increased abundance of p62 and colocalization of p62 and ubiquitinated proteins by ADP-ribosylcyclase inhibitor and CD38 gene silencing in podocytes

**Figure 6:**
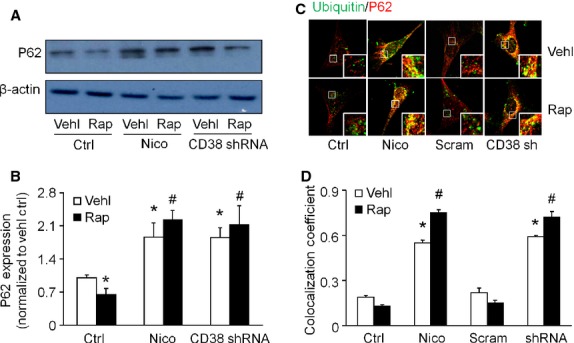
Reduced degradation of p62 and colocalization of p62 and ubiquitinated proteins in podocytes after CD38 inhibition. Podocytes were cultured for 24 hrs with 100 nM rapamycin in the absence or presence of 6 mM nicotinamide or CD38 shRNA transfection. (A) Analysis of p62 in whole cell lysates determined by immunoblotting. (B) Densitometric quantitation of p62 levels. (C) Typical confocal microscopic overlay images of p62 (Alex555, red colour) and ubiquitinated protein (Alex488, green colour)-stained podocytes. (D) Summarized data showing the colocalization coefficient of p62 and ubiquitin. Ctrl: Control; Vehl: Vehicle; Rap: rapamycin; Nico: Nicotinamide. Data are representative of six separate experiments and expressed as mean ± SEM. * Significant difference (*P* < 0.05) compared to the values from control group, ^#^ Significant difference (*P* < 0.05) compared to the values from rapamycin only group.

Inhibiting CD38 function by nicotinamide or CD38 shRNA transfection not only prevented p62 depletion but caused accumulation of p62 in rapamycin-treated cells (Fig. 6A and B). In contrast, either nicotinamide or CD38 shRNA transfection increased the colocalization of p62 and ubiquitin in untreated cells as well as in rapamycin-treated cells (Fig. 6C and D). Together, these data suggest that CD38 function is required for the degradation of APs.

### Role of calcium signalling in maturation of autophagy in podocytes

**Figure 7:**
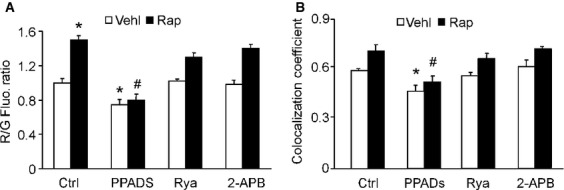
The effects of the CD38-regulated calcium pathway on the maturation of APLs in late autophagy in podocytes. Cells were cultured for 24 hrs with vehicle or 100 nM rapamycin in the absence or presence of 50 μM PPADS, 50 μM ryanodine, or 6.25 μM 2-APB after transient transfection of GFP-LC3 and RFP-Lamp1. (A) Flow cytometric analysis of the red/green fluorescence ratio. (B) Summarized data showing the colocalization coefficient of GFP-LC3 and RFP-Lamp1. Ctrl: Control; Rap: rapamycin; Ryr: ryanodine. Data are representative of six separate experiments and are expressed as mean ± SEM. * Significant difference (*P* < 0.05) compared to the values from control group, ^#^ Significant difference (*P* < 0.05) compared to the values from rapamycin only group.

The requirement for calcium signalling in the rapamycin-induced accumulation of APLs was investigated using inhibitors of three Ca^2+^ signalling messengers: IP3, NAADP, and cADPR. PPADS, an antagonist of the NAADP receptor, resulted in a significant decrease in acridine orange stained vesicles of either vehicle- or rapamycin-treated cells. However, neither 2-APB, an inhibitor of IP_3_ receptor, nor ryanodine, an inhibitor of cADPR receptor, had any apparent effect (Fig. 7A). Further confocal analysis also showed a selective effect of PPADS on colocalization of GFP-LC3 and RFP-Lamp1 in transduced podocytes (Fig. 7B). These results suggest that CD38 pathway involving NAADP-mediated calcium signalling is involved in APL accumulation and that a decrease in fusion of APs and lysosomes may be involved.

### Lysosome-dependent calcium release in response to an autophagic stimulus in podocytes is mediated by NAADP

**Figure 8:**
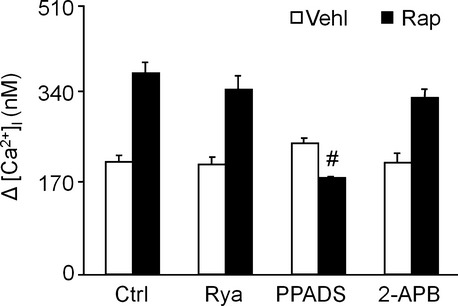
Calcium release from lysosomes in response to an autophagic stimulus in live podocytes. Podocytes were cultured for 24 hrs with vehicle or 100 nM rapamycin and then loaded with 10 μM Fura-2 as described under Materials and Methods. After treatment for 30 min. with 50 μM PPADS, 50 μM ryanodine, or 6.25 μM 2-APB, the [Ca^2+^]_i_ in response to 200 μM GPN was measured. Ctrl: Control; Rap: rapamycin; Ryr: ryanodine. The data are representative of six independent experiments and are expressed as the mean ± SEM. * Significant difference (*P* < 0.05) compared to the values from control group, ^#^ Significant difference (*P* < 0.05) compared to the values from rapamycin only group.

Rapamycin treatment significantly enhanced the GPN-induced lysosome-dependent Ca^2+^ release in podocytes (Fig. 8). This effect was blocked by PPADS but not by ryanodine or 2-APB. These results suggested that the rapamycin-induced lysosomal calcium release is mediated by the CD38 product, NAADP.

## Discussion

The major goal of the present study is to determine whether CD38-mediated regulation of lysosome function contributes to autophagic flux or autophagy maturation in podocytes. We found that lysosome function inhibitor bafilomycin and inhibition of CD38 by nicotinamide or CD38 gene silencing increased the APs formation, but decreased APLs content in podocytes under basal condition and following induction of autophagy by rapamycin. Moreover, our results showed that PPADS, an antagonist of NAADP receptor abolished the rapamycin-induced fusion of APs with lysosomes. Our results for the first time demonstrated a novel link between CD38 gene and the AP-lysosome degradation in podocytes.

We found a relatively high level of autophagy in podocytes even under basal conditions. Blocking the fusion of APs to lysosomes by bafilomycin led to the accumulation of APs upon rapamycin treatment, suggesting that rapamycin induces AP formation in podocytes. On the other hand, inhibiting AP formation by 3-MA resulted in a clear increase in markers of APLs in the rapamycin group (*P* = 0.064 *versus* non-rapamycin group), indicating that rapamycin might also be involved in the regulation of lysosome fusion and lysosome function. Therefore, the role of rapamycin in podocytes, besides potentially increasing AP formation, may also increase the number of functional lysosomes fusing with APs, thereby resulting in an overall enhancement of lysosome-mediated APs clearance. These results are in consistent with a previous study showing that rapamycin protected against MPTP-induced dopaminergic neurodegeneration by boosting lysosomal biogenesis, restoring the number of lysosomes, enhancing AP-lysosome fusion and increasing lysosome-mediated clearance of accumulated AP 31. Rapamycin is a potent non-nephrotoxic immunosuppressant that blocks the proliferation of T cells by inhibiting mTOR. Recent studies point to a complex effect of rapamycin on podocytes function, in which a context-dependent temporal and spatial fine tuning of mTORC1 activity seems to modify glomerular health and disease 32. However, the downstream signal of mTORC1 contributing to podocyte dysfunction is largely unknown. In this regard, it is possible that changes in autophagy may be a central factor contributing to this process. The induction of autophagy by rapamycin should be considered with great caution. A recent study has shown that rapamycin, an inhibitor of mTOR has two distinct effects on the autophagy process. Rapamycin initially activates autophagy, at later time-points its ability to inhibit mTOR actually blocks autophagy by preventing the reformation of lysosomes and APs which results in an accumulation of APLs 33.

In addition, our present study showed that AP formation in podocytes could be partly inhibited by 3-MA by blocking conversion of LC3-I to LC3-II; the fusion of AP to lysosome was blocked by bafilomycin through its inhibitory effect on the acidification of lysosomes. Autophagy inhibition further led to the accumulation of the endogenous autophagy substrate, p62, which suggests a decrease in degradation capacity in podocytes. Although 3-MA is a classic inhibitor of autophagy, 3-MA showed a small inhibitory effect in podocytes compared with other cells in which it shows strong effects such as neurons. These results highlight the differences in the control of autophagy in podocytes compared to other cell types and suggest that inducers or inhibitors of autophagy may be useful to ameliorate podocyte damage induced by autophagy dysfunction. Similar to neurons, our data and others 34 confirm that podocytes have a relatively high level of autophagy under basal conditions. Autophagy is a major mechanism to cope with stress in podocytes, and altered autophagy may abate the protective role or induce more serious results. For these reasons, it is necessary to understand mechanisms of autophagy regulation in podocytes.

Our laboratory 35–36 and others 37–38 have reported that the CD38 signalling pathway played an important role in the regulation of lysosome function. CD38 is a widely expressed mammalian ectoenzyme involved in many functions as diverse as cell proliferation and behaviour 39. The present study investigated the role of CD38 on the process of autophagy in podocytes and the underlying mechanism. The present study showed that silencing the CD38 gene *via* a CD38 shRNA or inhibiting its functional activity by ADP-ribosylcyclase inhibitor, nicotinamide, increased the level of LC3-II and caused the APs accumulation. However, the steady-state levels of LC3-II and AP reflect the net balance between the rates of formation and degradation of APs. Furthermore, we determined whether the increase in LC3-II and AP was caused by decreased lysosome degradation, using flow cytometry and immunofluorescence approaches we measured the efficiency of lysosome fusion with APs treated with either nicotinamide or CD38 shRNA. Our data in the present study showed that inhibition of CD38 gene expression or its enzymatic activity blunted the merging of lysosomes to APs. In addition, increased accumulation of P62 and ubiquitinated proteins also supported the conclusion that autophagy was attenuated by inhibition of CD38 gene. It may be speculated that CD38 serves as a ‘molecular switch’ in the regulation of autophagy under both physiological and pathological conditions. CD38 inhibition may impair the clearance of autophagic substrates and induce the podocyte damage.

Given the importance of lysosomes in autophagic degradation and the link between CD38 function and calcium release, we further examined the mechanisms by which altered CD38 signalling regulates lysosome function and control autophagy maturation. The present study demonstrated that, CD38-NAADP-calcium pathway played an important role in the fusion of lysosome with AP. In recent years, contradictory data have emerged to the role of cytosolic Ca^2+^ in autophagy, with both stimulatory and inhibitory effects on autophagy was reported. Hoyer-Hansen *et al*. 40 reported that Ca^2+^-mobilizing agents were potent inducers of autophagy. Dramatic increases in the number of APs were evident upon treatment with Ca^2+^-mobilizing stimuli such as vitamin D, ATP, thapsigargin, and ionomycin, which primarily control Ca^2+^ release from the ER. But Williams and colleagues had a different conclusion 41, suggested that autophagy was blocked by IP_3_R-dependent Ca^2+^ release from the ER into the cytosol *via* a calpain-dependent mechanism, and it was proposed that blocking IP_3_R increased autophagy in a manner that was independent of steady-state Ca^2+^ levels 41. Transient changes in intracytosolic Ca^2+^, which were affected by blocking the IP_3_R-specific Ca^2+^ oscillatory activity 42, might not result in decreased steady-state intracellular Ca^2+^ levels but could impact the activity of calpain and autophagy regulated by the Ca^2+^-calpain-Gsα-cAMP-IP_3_ pathway 43–44. Similarly, Sarkar *et al*. 45 found that autophagy increased at the level of lowered IP_3_, which was abrogated by PEI that increased intracellular levels of IP_3_. In addition, another report found that the inhibition of IP_3_R-mediated transient calcium flux was not necessary for the induction of autophagy 46.

It should be noted that the previous observations on calcium-regulated autophagy were mainly focused on Ca^2+^ release from the ER or the intracellular Ca^2+^. However, homotypic and heterotypic fusion events between organelles within the endolysosomal system that are required for trafficking are sensitive to Ca^2+^ release from lysosome 47, indicative of spatially restricted Ca^2+^ elevations in close proximity to the fusion machinery. Recently, the lysosome has emerged as one of the most important intracellular Ca^2+^ storage compartments, and the release of Ca^2+^ from the lysosome can be triggered by the calcium-mobilizing messenger, NADDP 48–49. Nicotinic acid adenine dinucleotide phosphate is the most powerful endogenous Ca^2+^-mobilizing compound known to date and is synthesized by the multi-functional signalling enzyme, CD38. It seems to invoke an initial release from acidic stores which is then amplified by Ca^2+^-induced Ca^2+^ release from the ER mediated by IP_3_R 50. Nevertheless, CD38 also plays a role in synthesis and regulation of cyclic ADP-ribose (cADPR). Cyclic ADP-ribose is derived from NAD, whereas NAADP is synthesized from NADP. The Ca^2+^ stores targeted by these mediators also differ; cADPR acts on the ER, similar to IP3, whereas NAADP targets the lysosome-like acidic stores. Alternatively, NAADP may regulate autophagy by local Ca^2+^ release events that promote fusion of the lysosome with AP. Our data in the present study showed that a significant decrease in APLs occurred in podocytes treated with PPADS, an antagonist of the NAADP receptor. However, 2-APB (an inhibitor of IP_3_ receptor) and ryanodine (an inhibitor of cADPR receptor) had no apparent effect on the numbers of late autophagic vesicles. These results suggest that the CD38/NAADP pathway may regulate autophagy by lysosome Ca^2+^ release events that promote fusion of the lysosome to AP. The exact mechanism of this novel signalling pathway of mammalian autophagy regulation remains to be determined. In conclusion, our results demonstrated that CD38-mediated regulation of lysosome function is involved in autophagy maturation in podocytes. Therefore, defect of this lysosome regulation of autophagy in podocytes may result in an impaired autophagy and ultimately progresses podocyte damage.

## Conflicts of interest

The authors confirm that there are no conflicts of interest.

## Supporting Information

**Figure S1.** CD38 gene silencing induced by CD38 shRNA transfection. **Figure S2.** Flow cytometric analysis of the red/green fluorescence ratio after acridine orange staining.
