# Supplementary material for: Autophagy maturation associated with CD38-mediated regulation of lysosome function in mouse glomerular podocytes
Source: J Cell Mol Med. 2013 Nov 17;17(12):1598–607. doi: 10.1111/jcmm.12173 (PMC3914646; doi:10.1111/jcmm.12173)
Supplement: Supplementary file 1 — Figure S1. CD38 gene silencing induced by CD38 shRNA transfection. Figure S2. Flow cytometric analysis of the red/green fluorescence ratio after acridine orange staining. [file jcmm0017-1598-sd1.doc]

**Supporting Information**

Supplemental Figure 1: CD38 gene silencing induced by CD38 shRNA transfection. Podocytes were transfected with CD38 shRNA and detected the CD38 expression by RT-PCR (A), Western blot analysis (B) and Immunohistochemical analysis (C). Scra: Scramble siRNA. Data are expressed as the mean ± SEM and are representative of six separate experiments. * Significant difference (*P*<0.05) compared to the values from scramble siRNA transfected group.

Supplemental Figure 2: Flow cytometric analysis of the red/green fluorescence ratio after acridine orange staining. Podocytes were starved for 90 min with EBBS solution in the absence or presence of 6 mM nicotinamide. Ctrl: Control, Vehl: Vehicle, Nico: Nicotinamide. Data are expressed as the mean ± SEM and are representative of six separate experiments. * Significant difference (*P*<0.05) compared to the values from control group, ^#^ Significant difference (*P*<0.05) compared to the values from starvation only group.
